# TRPM8 Regulates Mitochondrial Ca^2+^‐Dynamics, Temperature and Endoplasmic Reticulum‐Mitochondrial Contact Points in T Cell

**DOI:** 10.1111/jcmm.71014

**Published:** 2026-01-28

**Authors:** Tusar Kanta Acharya, Shamit Kumar, Tejas Pravin Rokade, Parnasree Mahapatra, Young Tae Chang, Chandan Goswami

**Affiliations:** ^1^ School of Biological Sciences National Institute of Science Education and Research Bhubaneswar Khurda Odisha India; ^2^ Training School Complex Homi Bhabha National Institute Mumbai India; ^3^ Department of Chemistry Pohang University of Science and Technology (POSTECH) Pohang Republic of Korea

**Keywords:** Ca^2+^‐buffering, immune regulation, immune synapse, metabolism, mitochondria, sub‐cellular organelle temperature, T‐cell activation

## Abstract

TRPM8 is a cold temperature‐sensitive and non‐selective Ca^2+^‐channel. Previously we have observed that TRPM8 is endogenously expressed and affects T cell activation process. Now, we report that TRPM8 regulates functions of mitochondria and ER, two important sub‐cellular compartments. Pharmacological modulation of TRPM8 and/or due to TCR‐treatment regulates mitochondrial Ca^2+^, ATP, membrane potential, cardiolipin level and mitochondrial temperature in a context‐dependent manner. In addition, TRPM8 alters the relative temperature of mitochondria and ER, ER‐mitochondrial contact points, mainly at the immunological synapse (IS), and thus TRPM8 has the potential to affect the overall cellular functions. Our data suggests both, i.e., the presence and enrichment of TRPM8 in the IS of T cells. We suggest that TRPM8 is a crucial regulator of Ca^2+^‐signalling in T cells and significantly contributes to Ca^2+^‐buffering by modulating cellular and sub‐cellular organelle functions. These findings are useful to understand the functions of T cells in different pathological conditions.

**Abbreviations**
BAPTA‐AM: cytosolic Ca^2+^‐chelator
CCCP: carbonyl cyanide m‐chlorophenyl hydrazone
ConA: concanavalin A
EGTA: extra cellular Ca^2+^‐chelator
ETY: ER Thermo Yellow
IS: immunological synapse
MAM: mitochondria‐associated endoplasmic reticulum membranes
MTY: Mito Thermo Yellow
NAO: Nonyl Acridine Orange
SERCA: sarcoplasmic/endoplasmic reticulum Ca^2+^‐ATPase
TRPM8: transient receptor potential cation channel subfamily M (melastatin) member 8

## 1 Introduction

Foreign antigens including viruses and bacteria are processed and presented to T cells by antigen‐presenting cells through major histocompatibility complexes. Antigen recognition leads to the formation of T cell synapses, which are made of a central supramolecular activation cluster (C‐SMAC) surrounded by a peripheral supramolecular activation cluster (P‐SMAC) [1]. Immediately after the formation of “immune synapse” (IS), intracellular Ca^2+^‐levels increase by the opening of calcium release‐activated calcium (CRAC/Orai 1) channels, leading to a cascade of cell signalling events important for T cell activation [2]. In order to fulfil the greater bioenergetic and biosynthetic demands caused by T cell expansion, cellular metabolism is highly regulated. Various metabolites modulate a wide range of cellular signalling that direct the differentiation of various T cell subsets [3]. The involvement of mitochondria in T cells seems to be crucial, as it not only provides the energy required for the activation process, but also performs Ca^2+^‐buffering and Ca^2+^‐signalling which are critical for activation [4, 5]. Mitochondrial metabolism is critical for the development of Treg cells and their functional maintenance, particularly oxidative phosphorylation and fatty acid oxidation, which maintain their immunosuppressive capabilities [6]. Studies on the control of innate immunity by mitochondria have received extensive research in recent years. It has been suggested that mitochondria are the primary nodes of the innate immune system [7]. Mitochondrial membrane potential, mitochondrial ROS and mtDNA are implicated in inflammasome activation. Inflammasome activation is linked to “pyroptosis”, a kind of inflammatory cell death mediated by mitochondria [8]. Mitochondria have the inherent ability to counter viral infection. MAVS is a mitochondrial outer membrane protein which activates NF‐kB and IRF‐3 signalling and induces the pro‐inflammatory cytokine IFN‐1 when responding to RNA viruses [8]. Discovery of MAVS (Mitochondrial Anti‐Viral Signalling) protein also indicates the critical role and evolutionary function of mitochondria in antiviral pathways too. Mitochondria are very dynamic organelles which play a crucial role in intracellular Ca^2+^‐buffering and energy mobilisation at specific cellular regions. It keeps the cellular Ca^2+^‐homeostasis stable by interacting with other organelles in a spatio‐temporal manner [9]. By abolishing the mitochondrial Ca^2+^‐uptake by CCCP, antimycin/oligomycin, in turn, decreases IS‐mediated intracellular Ca^2+^‐increment. Recently, it has been reported that during the process of T cell activation, mitochondria translocate to the IS [2]. After the formation of IS, T cells depend on nearby mitochondria for the supply of sufficient ATP for proliferation and clonal expansion [5]. The distribution of mitochondria to the IS becomes, therefore, essential for maintaining cellular Ca^2+^ and Ca^2+^‐mediated T cell activation [2]. The influx of Ca^2+^ from the extracellular source into the cytosol, which regulates several signalling pathways for T cell activation, is primarily facilitated by many Ca^2+^‐ion channels expressed over the plasma membrane [10].

Recent advancements in the investigation of ion channels in T cells indicate that, along with other channels, multiple TRP channels significantly contribute to T cell activation and functionality. TRP channels constitute a category of thermosensitive ion channels that play a role in cellular and sensory functions. Various subfamilies of TRP channels, including TRPV (Vanilloid), TRPM (Melastatin) and TRPA1 (Ankyrin) modulate intracellular Ca^2+^‐levels [11]. Recently, we investigated the expression and function of TRPM8 in T cells. By using pharmacological modulators of TRPM8, we observed that TRPM8 promotes TCR‐induced intracellular Ca^2+^ whereas inhibition of TRPM8 reduces the proliferation and cytokine release [12]. Accordingly, it has also been reported that truncated 4TM‐TRPM8 is localised in mitochondria and at the MAM (Mitochondria Associated Membrane) where it regulates mitochondrial Ca^2+^‐level [13]. In a recent study from our lab, we reported that TRPV4 localises to mitochondria and regulates mitochondrial physiology and ER‐mitochondrial contact points in resting and TCR‐treated T cells [14]. However, it is largely unknown if and how TRPM8 channel regulates MAM establishment and/or mitochondrial function in T cells. Here, we investigated the localisation of TRPM8 in the mitochondria and if pharmacological mediators regulating TRPM8 function can affect mitochondrial Ca^2+^‐signalling in murine CD3^+^ T cells. Here, we explored the possible changes in mitochondrial function and ER‐mitochondrial physiologies in T cells, especially in resting and in TCR‐treated conditions in response to TRPM8 modulation.

## 2 Material and Method

### 2.1 Isolation of Primary Murine T Cells

Isolation and culturing of T cells were conducted as previously described [12, 15]. All animal studies were carried out as per the approval from the IAEC (Institutional Animal Ethics Committee no: NISER/SBS/IAEC/AH‐245). Briefly, spleen from 4 to 6 week old male BALB/c mice was isolated and fragmented to derive a spleenocyte suspension. Dynabeads Untouched Mouse CD3^+^T cells Kit (Invitrogen) was used to isolate CD3^+^T cells from spleenocytes according to the manufacturer's guidelines. Primary T cells were cultured with RPMI (GIBCO) media supplemented with 10% Fetal Bovine Serum (GIBCO) in a humidity‐controlled incubator at 37°C and 5% CO_2_. For T cell expansion and activation, Dynabeads Mouse T‐Activator CD3/CD28 (Invitrogen) was used as per the manufacturer's instruction. After 12 h of T cell activation, clearly observable T cell blasts were developed.

### 2.2 Pharmacological Stimulation of TRPM8, Live‐Cell Confocal Microscopy and Image Quantification

The modulation of TRPM8 was done using agonist WS12 (Tocris) or Menthol (Sigma) and antagonist AMTB (Tocris). WS12 and Menthol were used at concentrations of 5 μM and 50 μM, respectively, whereas AMTB was used at 10 μM for 1 h unless stated otherwise. For live‐cell microscopy, Olympus FV3000 confocal microscope (60× objective with 1.35 NA, UPlanSApo) was used. All live‐cell imaging experiments were performed using a temperature and CO_2_‐controlled chamber made by OKOlab. Image analysis and quantification were carried out by using ImageJ. In all these cases, intensity per unit area was quantified using the ‘Measure’ function of ImageJ.

### 2.3 Detection of Cellular and Mitochondrial Ca^2+^

To measure cellular Ca^2+^‐levels, cells were labelled with Fluo4 AM (1 μM, 30 min) dye just before live cell imaging. For imaging of mitochondrial Ca^2+^, T cells were labelled with Rhod‐2‐AM (5 μM, 1 h). Cells were washed with media and incubated in fresh media for 1 h to minimise signal of Rhod‐2‐AM in the cytosol. Subsequently, cells were processed for live cell imaging. For instantaneous mitochondrial Ca^2+^‐imaging, the Rhod‐2‐AM labelled live cells were subjected to treatment with either TRPM8‐specific activator WS12 (5 μM) or Menthol (50 μM) at the 20th frame followed by time series recording for up to 400 frames (Total duration is ~7 min). For some experiments, cells were pre‐treated with either EGTA (5 μM), or BAPTA‐AM (5 μM), or Thapsigargin (1 μM) for 1 h followed by live cell imaging.

### 2.4 Labelling and Measurement for Mito‐Thermo Yellow (MTY) and ER Thermo Yellow (ETY)

MTY and ETY dyes are used for detecting mitochondrial and ER temperature respectively as described previously [16, 17, 18, 19]. Resting or TCR‐treated primary T cells were seeded in a 35 mm glass bottom dish (IBIDI) with either WS12, Menthol, or AMTB for 1 h. Both, MTY and ETY were used at a concentration of 0.5 μM for the last 15 min. Thereafter, cells were washed and prepared for live cell imaging. The fluorescence excitation and emission of MTY and ETY were determined at 530 nm/570 nm and 560 nm/585 nm respectively. The fluorescence intensity of MTY and ETY are inversely proportional to mitochondrial and ER temperature [18, 19]. Changes in the temperature (as derived from the changes in the fluorescence intensity of individual cells labelled with these thermal probes) are interpreted as a “relative change” with respect to the control condition.

### 2.5 JC1 and MitoSOX Labelling

Ratiometric JC‐1 (Invitrogen) and MitoSOX (Invitrogen) dyes were employed for the assessment of mitochondrial membrane potential and mitochondrial superoxide (mtROS) analysis respectively. Resting and TCR‐treated T cells were incubated with WS12, Menthol or AMTB for 1‐h, followed by the addition of dyes. Primary murine CD3^+^T cells were labelled with either JC‐1 (5 μM, 1‐h) or MitoSOX (2.5 μM, 30 min). Subsequently, the cells were washed with warm media, and live cell imaging was performed. JC‐1 dye has fluorescence emission characteristics that shift from green to red (525 nm to 590 nm) range. The fluorescence excitation and emission spectrum of MitoSOX was 510/580 nm.

### 2.6 ATP‐Red and Nonyl Acridine Orange (NAO) Labelling

ATP‐Red dye was used for mitochondrial ATP‐labelling as described previously [20]. The fluorescence intensity of the dye increases in direct proportion to the ATP‐level, and was measured at 510 nm (Ex). Murine T cells were seeded on a 35 mm glass bottom dish. Following 1‐h treatment with pharmacological agents, the cells were stained with ATP‐Red dye (5 μM). For the detection of cardiolipin, cells were labelled with NAO (Invitrogen, 0.5 μM) as described before [21]. An elevation in NAO intensity correlates with an increase in the cardiolipin levels. Following labelling, the cells were washed and taken for live cell imaging.

### 2.7 Analysis of ER‐Mitochondrial Contact Sites

Resting and TCR‐activated T cells were treated with modulators of TRPM8 for 1 h. For labelling of ER, ER‐Tracker Blue‐White DPX (0.5 μM, Invitrogen) was used, whereas for mitochondria, MitoTrackerRed CMXRos (0.5 μM, Invitrogen) was used for 30 min. The quantification of ER‐mitochondrial contact sites was conducted as previously described [12, 22]. In brief, images were converted into binary, total ER and total mitochondrial particles of individual cells were calculated using the “Analyse Particle” function in ImageJ. For colocalisation analysis, “co‐localisation” plugin of ImageJ was utilised and subsequently total colocalized particles were also calculated for respective cells using the “Analyse Particle” function. Values of total ER, total mitochondria and total colocalized pixels from multiple cells were aggregated together and plotted using GraphPad Prism 8.

### 2.8 Immunofluorescence Staining

For immunofluorescence analysis, resting T cells were incubated with MitoTrackerRed CMXRos (0.5 μM) for 30 min and subsequently fixed with 4% PFA. TCR‐treated T cells were fixed by 4% PFA, washed thrice with 1× PBS and permeabilized by using 0.1% TritonX‐100 in PBS (for 5 min). Subsequently, cells were blocked by Bovine Serum Albumin (BSA, 5%) for an hour. Cells were then incubated overnight at 4°C with anti‐TRPM8 (Alomone; Cat#ACC‐049) and anti‐OPA1 (Novus; Cat#NBP1‐71656) primary antibodies at 1:500 and 1:100 dilutions respectively. Subsequently, Alexa flour‐labelled anti‐Rabbit (1:500) (Invitrogen) and anti‐Mouse (1:200) (Invitrogen) secondary antibodies were used for 1 h. Further, cells were rinsed with 1× PBS thrice and confocal imaging was performed.

### 2.9 Super Resolution Imaging

The super resolution microscopy was performed with Zeiss Elyra7 super resolution microscope with lattice SIM^2^. Naïve CD3^+^ T cells are labelled with the MitoTrackerRed CMXRos and fixed and further immunostained with TRPM8 antibody. Also, fixed cells were double labelled for TRPM8 and mitochondrial membrane protein OPA1 as described above. All images are processed with the Zeiss Zen blue and Zeiss Zen black software. Brightness and contrast of the images were adjusted for optimum representation of the images.

### 2.10 Statistical Test

Statistical analysis and *p* values were calculated by using GraphPad Prism 8 (Version 8.00). Data are represented in the graphs as mean ± SEM. One‐way was performed for comparison of more than 2 groups. Turkey's multiple comparison test was done to compare the mean of different groups. Student's *t*‐test was performed for comparison of only two groups. The **** symbol represents *p*‐value ≤ 0.0001; *** symbol represents *p* value ≤ 0.001; ** symbol represents *p*‐value between 0.001 and 0.01; * symbol represents *p*‐value between 0.01 and 0.05; and “ns” (non‐significant) represents *p* value > 0.05.

## 3 Results

### 3.1 TRPM8 Is Endogenously Expressed in the Plasma Membrane as Well as in Mitochondria

**FIGURE 1:**
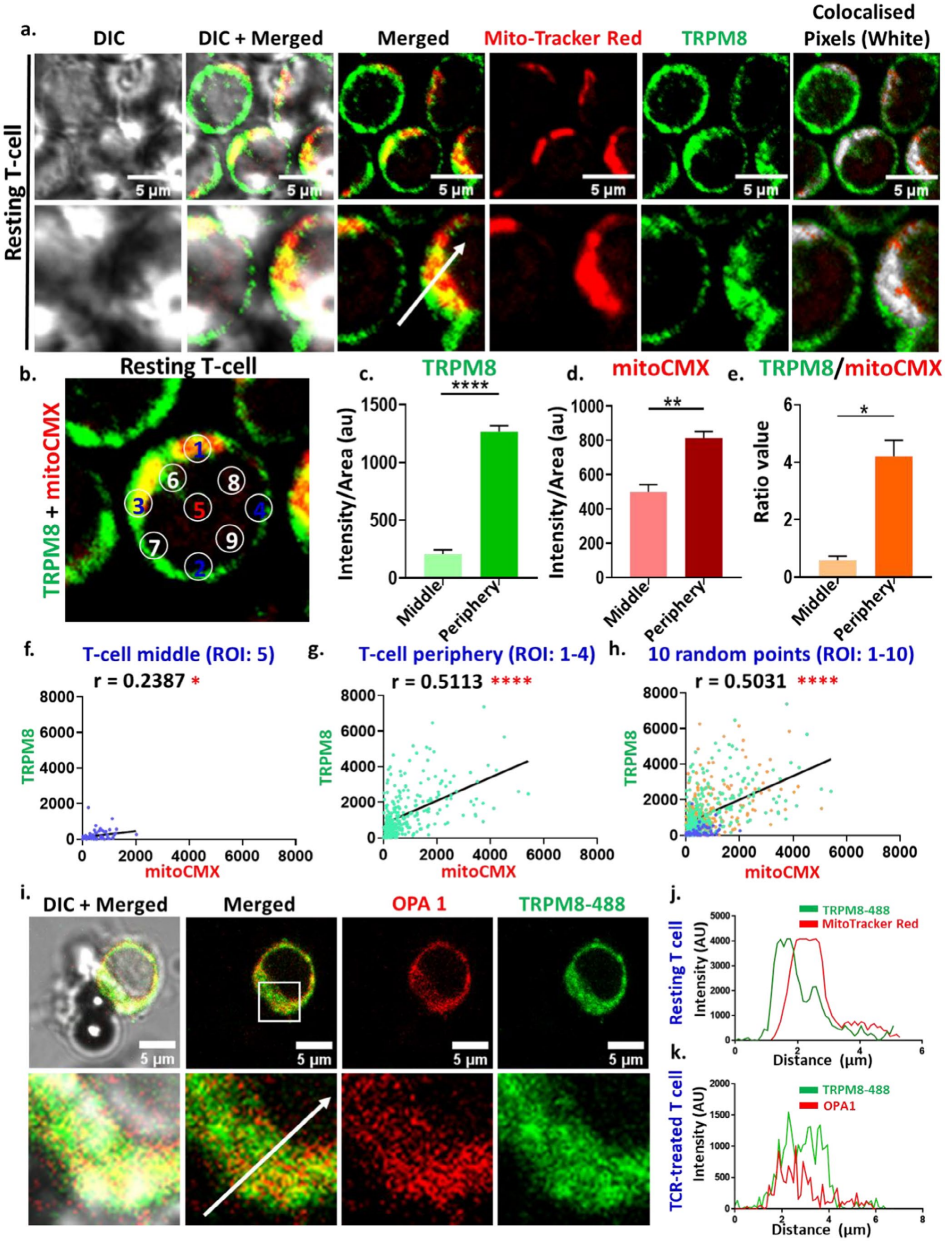
Endogneous TRPM8 localises with mitochondria in primary T cell. (a) Representative image demonstrating the colocalization of TRPM8 with Mito‐Tracker Red in resting T cells. (b–e) Shown is the confocal image of T cell indicating the distribution pattern of TRPM8 in mitochondria of T cells. Quantitative analysis of 10 different ROI's from more than 70 cells suggests enrichment of both TRPM8 and mitochondria in the periphery of T cells. Students *t*‐test; *p < 0.05, **p < 0.01, ****p < 0.0001. (f–h) Correlation analysis suggests that both TRPM8 and mitochondria positively correlates in T cells indicating possible presence of TRPM8 in mitochondria. (i) Shown are the representative images of TCR‐treated T cells immuno‐stained for mitochondrial marker OPA‐1 and TRPM8. (j–k) The intensity profile across the white line indicates the colocalization between TRPM8 and Mito‐Tracker Red/OPA1 in resting and TCR‐treated T cell.

We previously reported the presence of TRPM8 in T cell [12]. The localization of 4TM‐TRPM8 (a truncated version) in mitochondria and Mitochondria‐Associated Membrane (MAM) has been established [3]. However, the presence of full‐length 6TM‐TRPM8 in mitochondria in T cells has not been demonstrated. In this study, we investigated the mitochondrial localization of TRPM8 both in resting and in TCR‐stimulated CD3^+^ T cells. To assess this, we employed two distinct mitochondrial markers, i.e., MitoTracker Red and OPA1 in combination with TRPM8 immunostaining. Co‐staining revealed discrete colocalization points between TRPM8 and mitochondrial markers (indicated by yellow regions) under both resting and TCR‐stimulated conditions (Figure 1a,i). Notably, the degree of colocalization varied among individual T cells, suggesting heterogeneity in TRPM8 distribution.

Next, we examined region‐specific localization of TRPM8 in T cells. Fluorescence intensity of TRPM8 and MitoTracker Red was quantified across the central, peripheral and randomly selected regions of T cells (Figure 1b). It depicts that the expression/localization of TRPM8, mitochondria and TRPM8/mitoCMX ratio are higher at the peripheral region of the T cells (Figure 1c–e). Correlation analysis of TRPM8 and mitoCMX intensities across 10 different regions showed a stronger correlation at the periphery (*r* = 0.5) as compared to the central region (*r* = 0.2) (Figure 1f–h). This indicates that the TRPM8 level positively correlates with mitochondrial localization in the peripheral region of the T cells.

### 3.2 TRPM8 Colocalizes With Mitochondrial Markers

**FIGURE 2:**
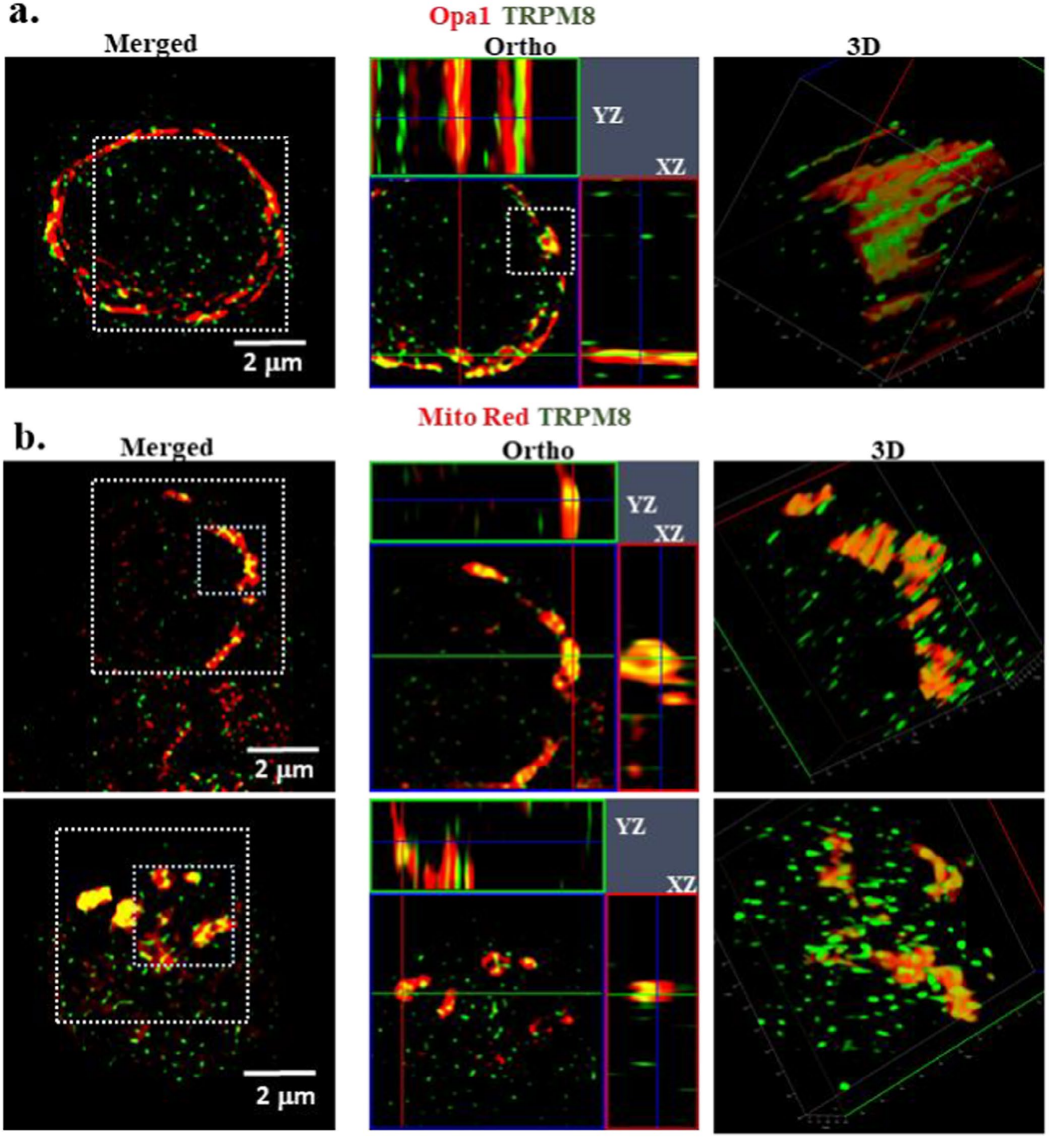
TRPM8 is present in the mitochodria‐enriched regions of T cell. Shown are the images aquired with Lattice SIM^2^ on super resolution microscope. (a, b) Naïve CD3^+^ T cells were labelled with TRPM8 (Green) and mitochondrial markers (Red), namely Opa1 (a) and MitoTrackerRed CMXRos (b) and subject to super resolution imaging. Scale bar in 2 μm in each case. TRPM8 is present in Opa1‐enriched mitochondrial structures or MitoTrackerRed CMXRos enriched structures Enlarged areas (indicated by white dotted lines) are shown in orthogonal view (middle panel). Much smaller regions (indicated by coloured dotted line) are also shown in 3D mode for better depiction.

In order to explore the possible association or even localization of TRPM8 in mitochondria, we performed super resolution microscopy with T cells that are double labelled with TRPM8 and mitochondrial markers. The detailed SIM^2^ image analysis suggests that TRPM8 colocalizes with the mitochondrial markers (Figure 2a,b). TRPM8 enrichment was seen in areas that have more mitochondria or clusters of mitochondria. Also, the colocalization between OPA1 and TRPM8 suggests that TRPM8 is likely to be present in subsets of mitochondria.

### 3.3 TRPM8 Regulates Both Mitochondrial Ca^2+^‐Levels and Ca^2+^‐Dynamics

**FIGURE 3:**
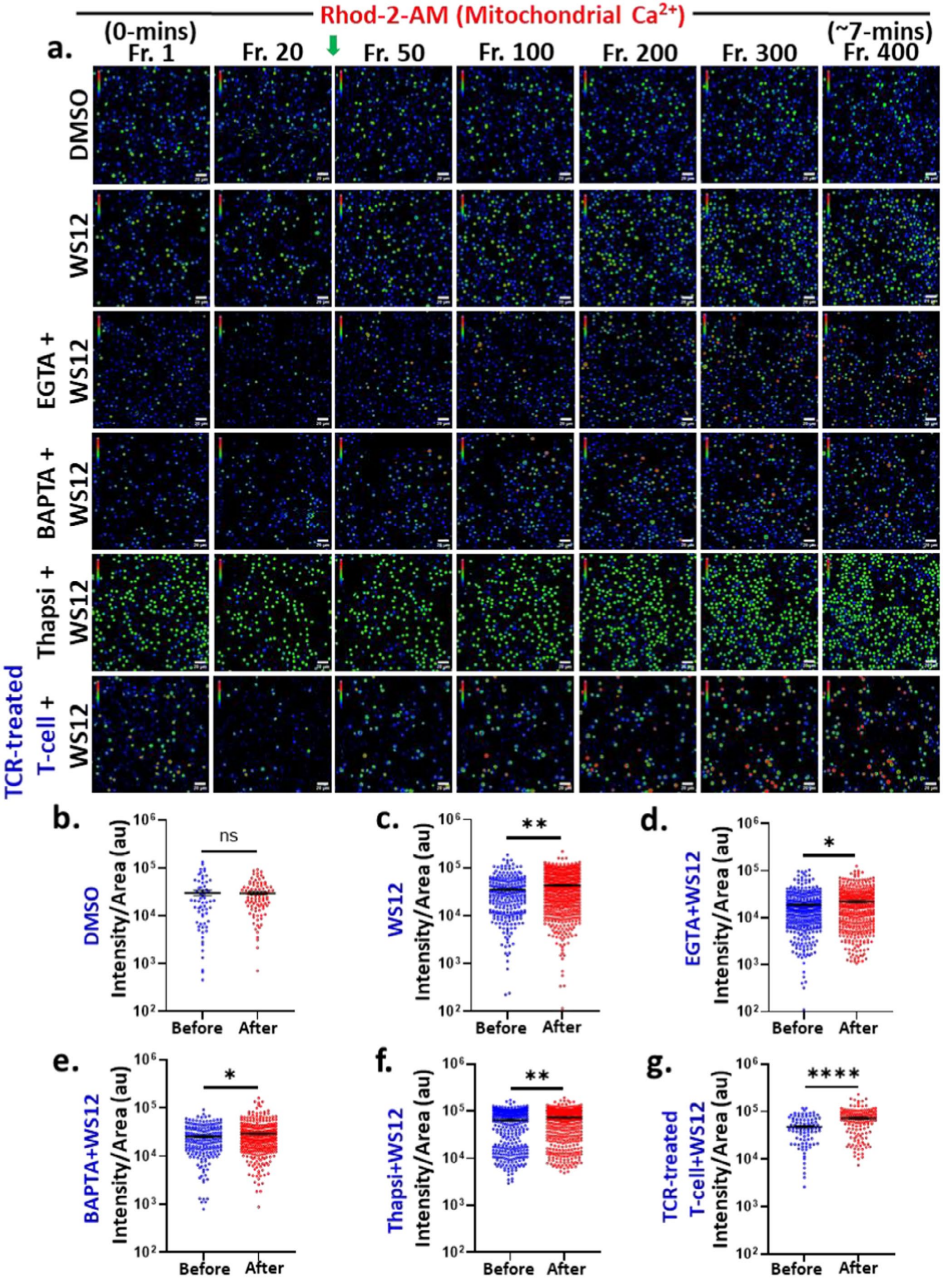
TRPM8 activation by specific synthetic activator increases mitochondrial Ca^2+^level instantly in murine CD3^+^ T cells. (a) Shown are the representative images acquired from the time‐series mitochondrial Ca^2+^‐imaging in primary murine CD3^+^ T cells represented in rainbow RGB intensity scale (blue for low to red for high intensity value). (b) DMSO addition does not alter the mitochondrial Ca^2+^‐level of T cells. (c) Addition of TRPM8‐specific agonist WS12 (5 μM) resulted in instantaneous mitochondrial Ca^2+_^influx in T cells. (d–f) Cells were pre‐incubated with EGTA (extracellular Ca^2+^‐chelator, 5 μM), BAPTA‐AM (cytosolic Ca^2+^‐chelator, 5 μM) and Thapsigargin (SERCA‐inhibitor and ER Ca^2+^‐chelator, 1 μM) for 1 h. WS12 addition results in an instant increment in mitochondrial Ca^2+^‐level in all the pre‐treated conditions but with different extent. (g) Activation of TRPM8 results in rise in mitochondrial Ca^2+^ of TCR‐treated T cells also. Scale bar is 20 μm. Fluorescence intensities of individual cell from initial frame and final frame of the time series experiment are represented. (*n* ≥ 100) cells in each condition were quantified. Students *t*‐test; ns = non‐significant, *p < 0.05, **p < 0.01, ****p < 0.0001.

Mitochondrial Ca^2+^‐homeostasis plays important roles in T cell activation, proliferation and different functions [5, 23]. Activation of TRPM8 by synthetic (WS12) and natural (Menthol) agonist has been shown to elevate cytosolic‐Ca^2+^ in T cells [12]. In this study, we monitored the immediate changes in mitochondrial Ca^2+^ (based on fluorescence of Rhod‐2AM) upon pharmacological activation of TRPM8 in different cellular conditions (Figure 3a). Tracking individual T cells over the duration of the time series experiment (~7 min) was challenging due to non‐adhesion (their motile/floating characteristics). For this purpose, the fluorescence in the initial time point (before stimulation) is considered as the reference while the fluorescence intensities at the end of the experiment (after stimulation) of the time series were quantified. DMSO‐treatment had no significant effect on mitochondrial Ca^2+^‐levels (Figure 3b). However, stimulation with WS12 in resting T cells induced a rapid and marked increase in mitochondrial Ca^2+^ (Figure 3c). Given that mitochondrial Ca^2+^ is significantly influenced by both the cytosol and ER, we further investigated the alterations in mitochondrial Ca^2+^‐levels by treating the cells with EGTA (extra‐cellular Ca^2+^‐chelator), BAPTA‐AM (used as a cytosolic Ca^2+^‐chelator) and Thapsigargin (used as a SERCA‐inhibitor that depletes ER Ca^2+^) for 1 h. We noted that mitochondrial Ca^2+^‐levels rise in all these pre‐treated conditions, albeit with various extents, suggesting that TRPM8‐mediated mitochondrial Ca^2+^‐influx in T cells relies on multiple Ca^2+^‐sources including cytosolic, extracellular and ER‐derived sources (as blocking any of these sources does not abolish the mitochondrial Ca^2+^‐increment) (Figure 3d–f). A comparable pattern was observed in TCR‐stimulated T cells, where WS12 also induced a significant mitochondrial Ca^2+^ increase (Figure 3g).

**FIGURE 4:**
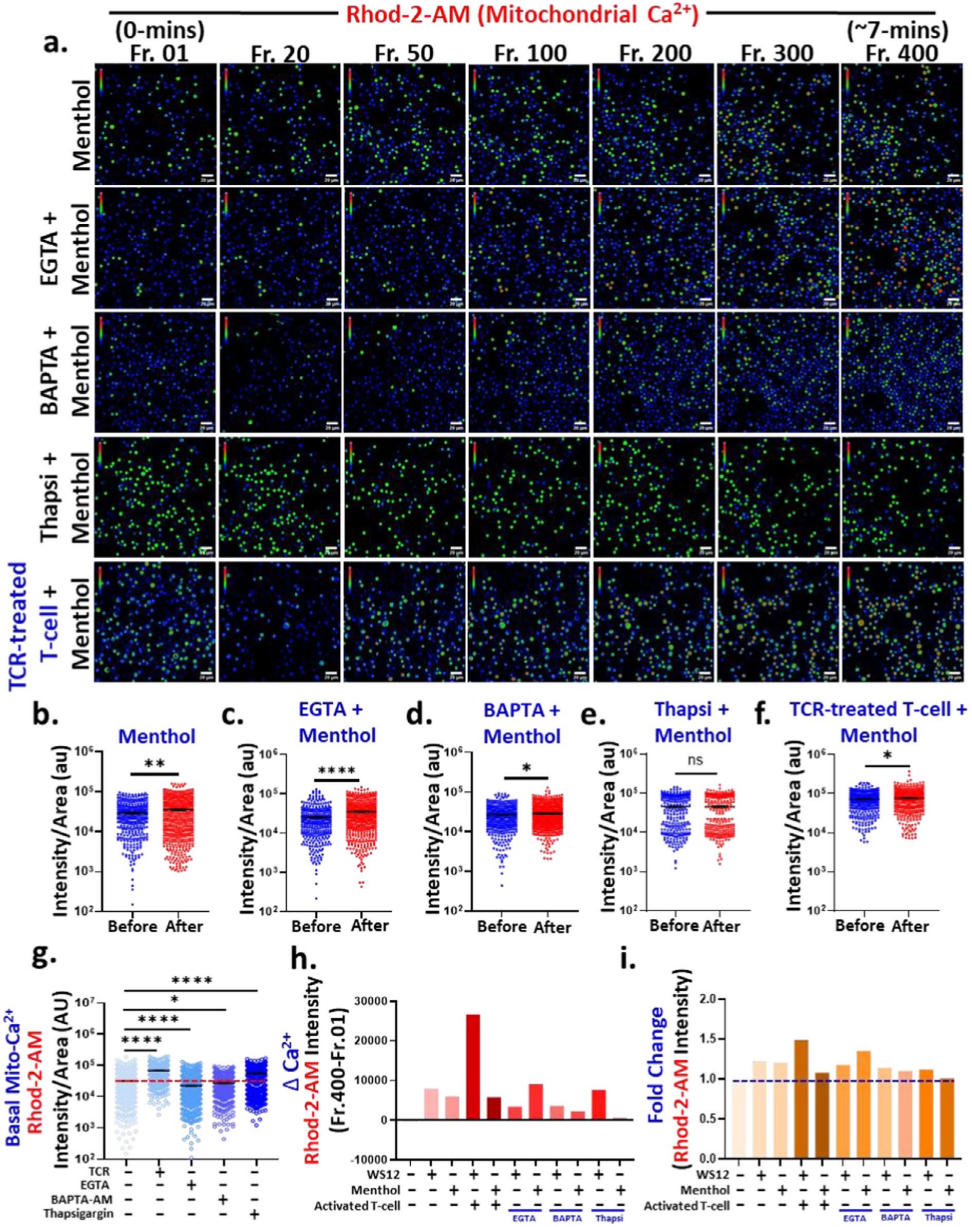
TRPM8 activation by Menthol (a natural agonist) increases mitochondrial Ca^2+^‐status in murine CD3^+^ T cells. (a) Shown are the representative images (represented in intensity scale coded blue as low to red as high) acquired during time series mitochondrial Ca^2+^‐imaging in murine CD3^+^ T cells. Fluorescence intensities of individual cell from initial frame and final frame of the time series experiment were quantified. (*n* ≥ 100) cells in each condition were quantified. Scale bar is 20 μm. (b) Mitochondrial Ca^2+^‐level increase upon addition of natural TRPM8 agonist Menthol (50 μM) at the 20th frame in resting T cells. (c–e) Cells were incubated with EGTA (extracellular Ca^2+^‐chelator, 5 μM), BAPTA‐AM (cytosolic Ca^2+^ chelator, 5 μM) and Thapsigargin (SERCA‐inhibitor and ER Ca^2+^‐chelator, 1 μM) for 1‐h. Addition of Menthol causes instantaneous mitochondrial Ca^2+^‐influx. (f) In TCR‐treated cells also, Menthol increases the mitochondrial Ca^2+^‐level. Students *t*‐test; ns = non‐significant, *p < 0.05, **p < 0.01, ****p < 0.0001. (g) Basal mitochondrial Ca^2+^ in different conditions were quantified and TCR‐treated cells have higher basal level of Ca^2+^. One‐way ANOVA; *p < 0.05, ****p < 0.0001. (h) Fold‐change in mitochondrial‐Ca^2+^ compared to control condition were quantified. (i) Delta change in Ca^2+^ (final intensity‐initial intensity) in different conditions was calculated. *n* ≥ 100 cells in each condition.

We next conducted parallel experiments using Menthol. In resting T cells, Menthol treatment led to an increase in mitochondrial Ca^2+^‐levels (Figure 4b). This effect remained significant under EGTA and BAPTA‐AM pre‐treatment (Figure 4c,d). However, in Thapsigargin pre‐treated cells, Menthol failed to elevate the mitochondrial Ca^2+^‐level (Figure 4e), indicating a key role for ER‐derived Ca^2+^. Notably, the rate of mitochondrial Ca^2+^ increase under BAPTA‐AM and Thapsigargin pre‐treatment was lower than that seen in EGTA‐treated cells, underscoring the importance of store‐operated Ca^2+^‐sources in Menthol‐induced responses. Menthol also triggered a rapid mitochondrial Ca^2+^‐influx in TCR‐treated cells (Figure 4f).

Subsequently, we quantified the basal mitochondrial Ca^2+^‐concentrations (Rhod‐2‐AM intensity at Frame 0) across all pretreatment conditions. Initially, we noted that TCR‐treated and Thapsigargin pre‐treated T cells exhibit significantly elevated levels of basal mitochondrial Ca^2+^ in comparison to resting T cells (Figure 4g). In contrast, cells pre‐treated with EGTA and BAPTA‐AM exhibit a reduced level of basal mitochondrial Ca^2+^ (Figure 4g). We assessed the alteration in the Rhod‐2‐AM intensity upon stimulus addition (Frame 400–Frame 0) (Figure 4h). Addition of WS12 triggers more instantaneous mitochondrial Ca^2+^‐influx as compared to Menthol in resting T cells. In TCR‐treated cells, WS12 triggered the largest Ca^2+^‐increase, while Menthol elicited responses comparable to those in resting cells. These results highlight the enhanced sensitivity of activated T cells to TRPM8‐mediated mitochondrial Ca^2+^‐influx. In EGTA and BAPTA‐AM pre‐treated conditions, WS12 addition reduced mitochondrial Ca^2+^‐influx as compared to resting T cells. However, in Thapsigargin pre‐treated cells, the alteration in mitochondrial Ca^2+^ is similar to only WS12 stimulated cells indicating TRPM8‐specific increment in mitochondrial Ca^2+^‐level is dependent more on extracellular than ER or intracellular Ca^2+^‐sources. For Menthol, EGTA pre‐treatment led to slightly greater mitochondrial Ca^2+^ changes compared to Menthol alone, while BAPTA‐AM reduced this effect and Thapsigargin completely abolished it. These findings suggest that Menthol‐induced mitochondrial Ca^2+^‐influx is strongly ER‐dependent and partially reliant on intracellular Ca^2+^ stores. An identical pattern was also observed in the fold‐change in fluorescence intensity of Rhod‐2‐AM across all conditions (Figure 4i).

Overall, our data suggests that activation of TRPM8 (by both specific and natural agonists) results in an instantaneous influx of mitochondrial Ca^2+^, which is dependent on multiple Ca^2+^‐sources (such as extracellular, intracellular and ER). Also, TCR‐treated T cells exhibit elevated basal mitochondrial Ca^2+^ and TRPM8 plays an important role in maintaining that.

### 3.4 Modulation of TRPM8 Impacts the Basal Level of Mitochondrial‐ and Cytosolic Ca^2+^

**FIGURE 5:**
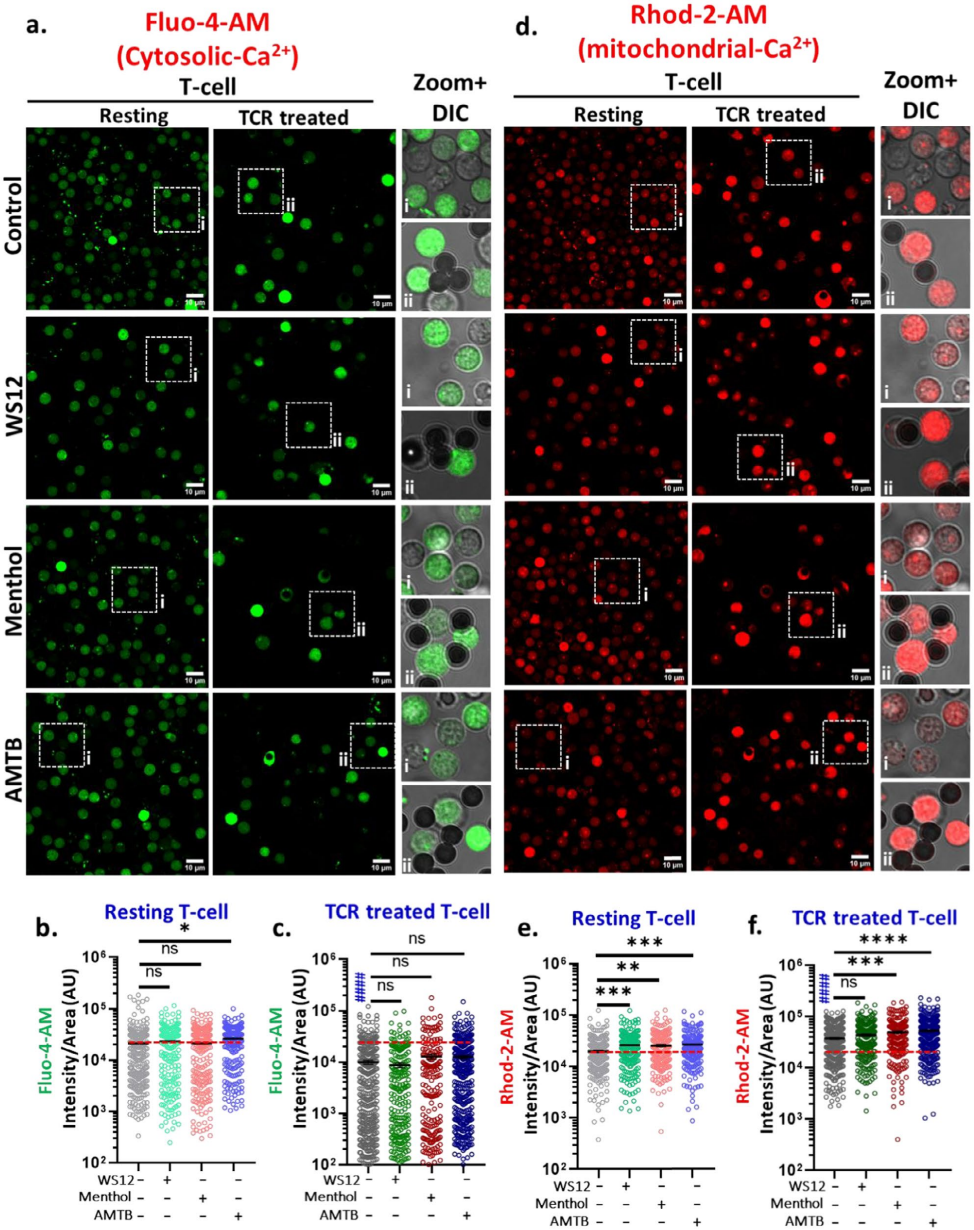
TRPM8 modulation increases basal cytosolic and mitochondrial Ca^2+^ in murine CD3^+^ T cells. (a–f) Representative images of basal cytosolic Ca^2^^+^ and mitochondrial Ca^2+^ in murine CD3^+^ T cells are shown. Resting and activated cells were treated with TRPM8 modulators for 1 h and simultaneously basal cytosolic Ca^2+^ was measured by Fluo4 AM and mitochondrial Ca^2+^ was measured by using Rhod‐2 AM. Scale bar is 10 μm. (b, c) and (e, f) Quantification of basal Fluo4 AM (Cytosolic Ca^2+^‐level) (b, c) and Rhod‐2 AM (mitochondrial Ca^2+^‐level) (e, f) fluorescence intensities in resting and TCR‐treated T cells (*n* ≥ 100 cells in each condition were quantified. One‐way ANOVA, *p < 0.05, **p < 0.01, ****p < 0.0001). # represents comparison between resting control cells and TCR‐treated control cells (student's *t*‐test, ^####^ *p* < 0.0001).

Mitochondria are recognised for their significant role in intracellular Ca^2+^‐mobilisation and their effective Ca^2+^‐buffering capabilities [4]. We investigated the impact of prolonged modulation of TRPM8 on the basal mitochondrial and cytosolic Ca^2+^‐levels, we treated both resting and TCR‐activated T cells with WS12, Menthol, or the TRPM8 inhibitor AMTB for 1 h. Subsequently, we simultaneously measured basal cytosolic and mitochondrial Ca^2+^‐levels in individual T cells using Fluo‐4 AM and Rhod‐2 AM, respectively (Figure 5a,d). In resting T cells, 1‐h activation of TRPM8 (with WS12 or Menthol) did not significantly affect cytosolic Ca^2+^‐levels. However, TRPM8 inhibition by AMTB led to a notable increase in cytosolic Ca^2+^ (Figure 5a,b). In contrast, TCR‐stimulated T cells show no significant changes in cytosolic Ca^2+^ concentration across any treatment condition (Figure 5a,c). We next assessed basal mitochondrial Ca^2+^‐level. In resting T cells, mitochondrial Ca^2+^‐levels rise due to the regulation of TRPM8 by chronic incubation with pharmacological modulators (Figure 5d,e). Furthermore, TCR‐treated T cells displayed a significantly higher basal mitochondrial Ca^2+^ concentration (~1.92‐fold increase) compared to resting cells (Figure 5d,f). In these activated cells, Menthol and AMTB further raise the mitochondrial Ca^2+^‐load (Figure 5d,f) while WS12 caused a small but non‐significant increase. Collectively, these results indicate that the prolonged modulation of TRPM8 influences the mitochondrial as well as cytosolic Ca^2+^‐load of T cells, both in resting and TCR‐treated conditions.

**FIGURE 6:**
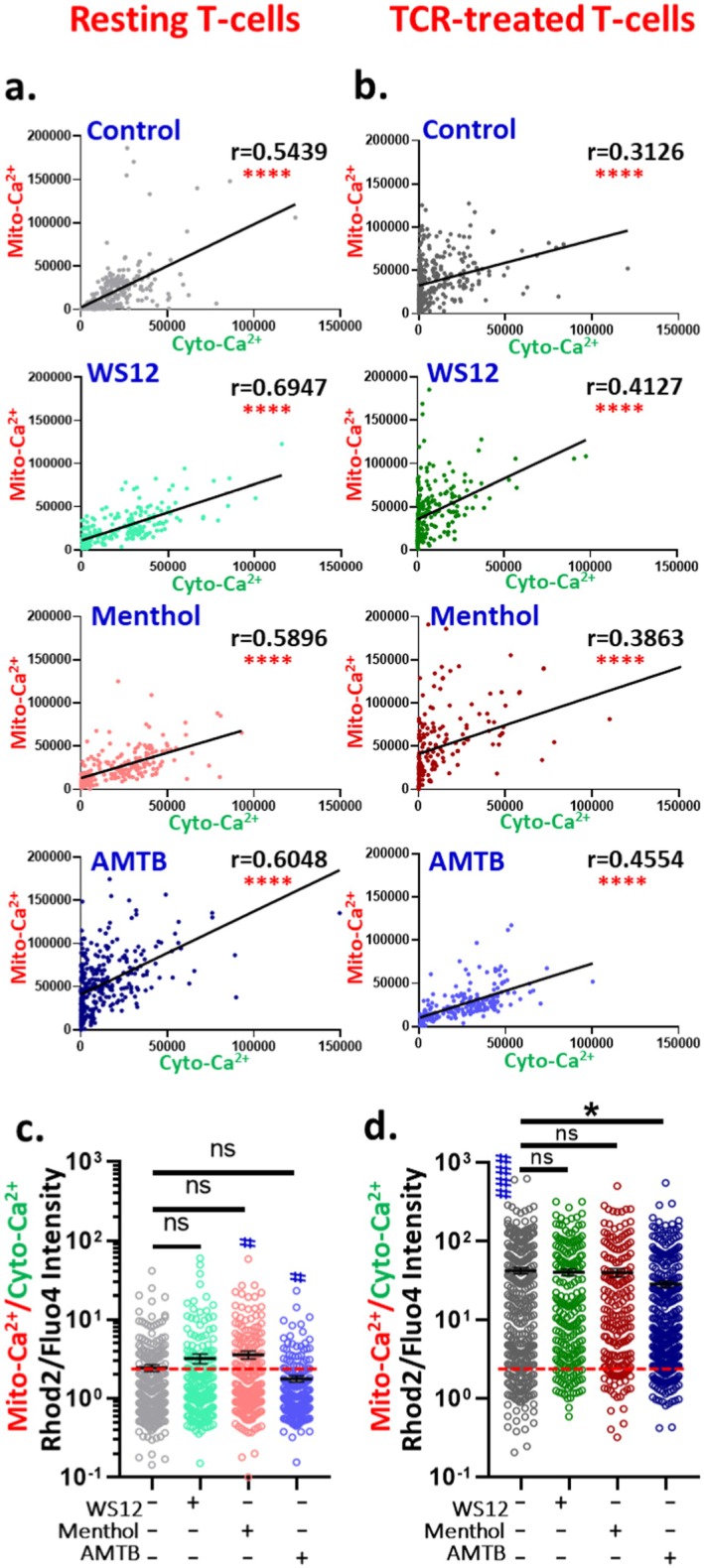
Correlation study between cytosolic Ca^2+^ and mitochondrial Ca^2+^. (a) In resting CD3^+^ T cells the correlation between cytosolic Ca^2+^ and mitochondrial Ca^2+^ in different experimental conditions is represented. In TRPM8 activated cells, the correlation is high whereas in AMTB‐treated cells the correlation is reduced. (b) The correlation between cytosolic Ca^2+^ and mitochondrial Ca^2+^ in TCR‐treated CD3^+^ T cells is represented. In TCR‐treated cells, the trend is opposite, TRPM8‐activated cells have less correlation but inhibited cells have high correlation. (c, d) graph represents the ratio of mitochondrial Ca^2+^‐intensity and cytosolic Ca^2+^‐intensity in resting as well as activated conditions. The ratio is high in TCR‐treated cells as compared to resting cells. In both conditions (resting as well as in TCR‐treated conditions), AMTB reduces the spontaneous activity of TRPM8 causing reduced mitochondrial Ca^2+^/Cytosolic Ca^2+^‐ratio. (*n* ≥ 100 cells in each condition were quantified, One‐way ANOVA, *p < 0.05). # represents comparison with resting control cells (student's *t*‐test, ^#=^ *p* < 0.05, ^####^ *p* < 0.0001).

To examine the interaction between mitochondrial Ca^2+^ and cytosolic Ca^2+^, we analysed the ratio of mitochondrial to cytosolic Ca^2+^ (mito‐Ca^2+^/cyto‐Ca^2+^) for individual T cells under the various treatment conditions. In resting T cells, TRPM8 activation (by WS12 and Menthol) marginally increased this ratio compared to untreated controls (Figure 6a). In the case of TCR‐treated T cells, the mitochondrial‐Ca^2+^/cytosolic‐Ca^2+^ ratio increases (~17.5‐fold) relative to resting T cells (Figure 6b). While TRPM8 activation did not significantly alter this ratio in TCR‐treated cells, TRPM8 inhibition (by AMTB) significantly reduced it, suggesting that TRPM8 helps to maintain elevated mitochondrial Ca^2+^ relative to the cytosol in activated T cells (Figure 6b). We further examined the dependency between mitochondrial Ca^2+^ and cytosolic Ca^2+^ using correlation analysis across all treatment groups. In resting T cells, a moderate positive correlation (*r* > 0.5) was observed under all conditions (Figure 6c). Notably, both TRPM8 activation and inhibition increased the correlation coefficient, indicating an enhanced interdependence between mitochondrial and cytosolic Ca^2+^ under TRPM8‐modulated conditions. This may also indicate that in these conditions, mitochondrial Ca^2+^‐buffering capacity is low, especially with respect to cytosolic Ca^2+^. In TCR‐treated T cells, this correlation declined (*r* = 0.3126) compared to resting cells (r = 0.5439), likely reflecting altered Ca^2+^‐buffering dynamics upon activation (Figure 6d). However, TRPM8 modulation in TCR‐treated cells again increased this correlation, supporting the idea that TRPM8 regulates intracellular Ca^2+^ distribution and buffering. A similar pattern was observed, indicating that modulation of TRPM8 (both activation and inhibition) enhances the correlation value, suggesting that increased TRPM8 modulation strengthens the interdependence of mitochondrial‐Ca^2+^ and cytosolic‐Ca^2+^. Together, our findings suggest that TRPM8 modulates the mitochondrial Ca^2^ and cytosolic Ca^2+^‐levels in T cells and affects the sub‐cellular Ca^2+^‐buffering by regulating the ratio of mitochndrial‐Ca^2+^ to cytosolic‐Ca^2+^.

### 3.5 TRPM8 Regulates ATP Production

**FIGURE 7:**
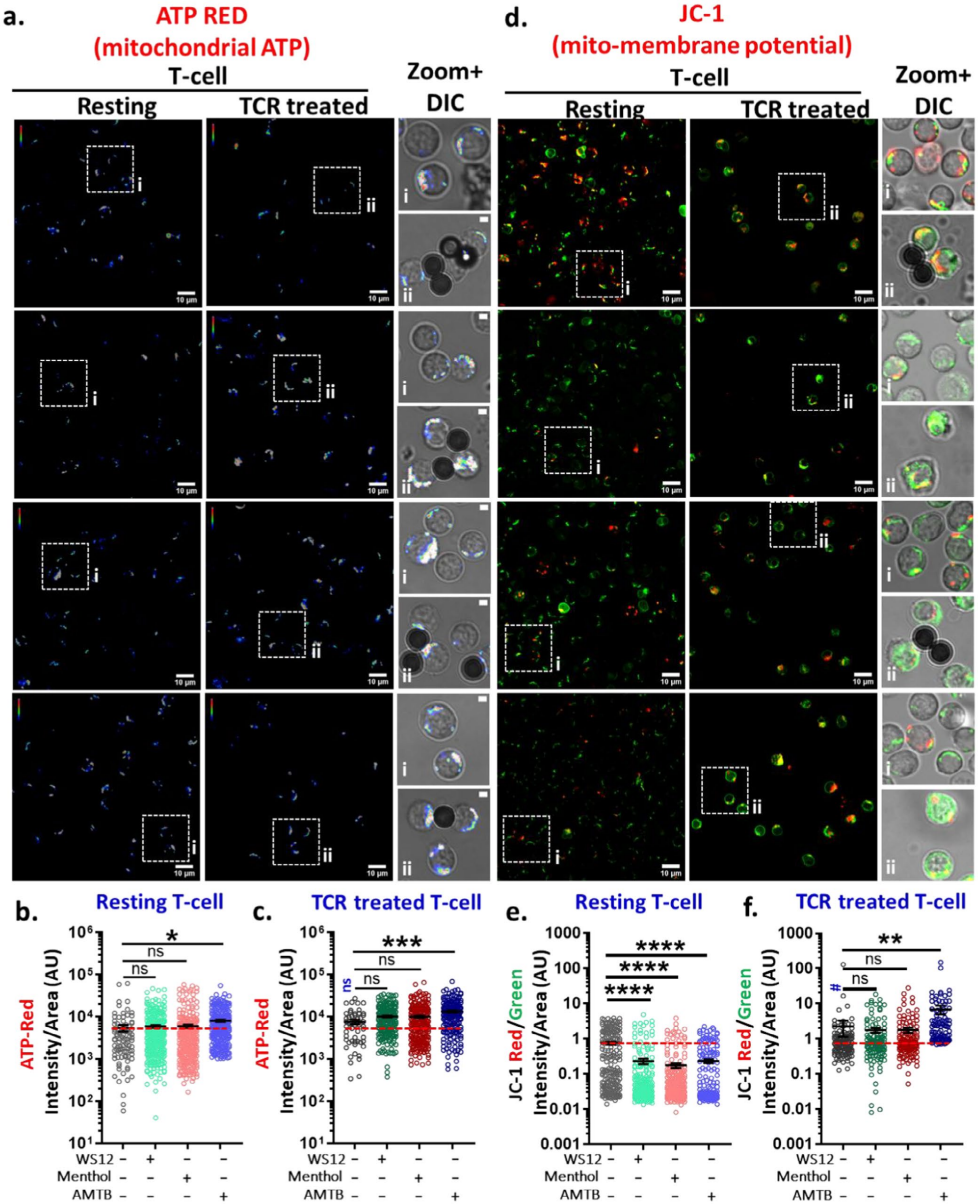
TRPM8 regulates mitochondrial ATP and membrane potential. (a–c) Murine CD3^+^ T cells at resting and TCR‐treated condition were treated with TRPM8 agonist WS12 (5 μM), Menthol (50 μM) and TRPM8 antagonist AMTB (10 μM). The mitohcondrial ATP level was measured by using ATP red dye (5 μM). ATP level is high in TCR‐treated cells as compared to resting cells. AMTB‐treated cells increase mitochondrial ATP level in both resting and activated T cells. Scale bar: 10 μm. (*n* ≥ 100 cells in each condition were quantified). (d–f) Both resting and TCR‐treated cells were treated with TRPM8 modulators and subsequently stained with JC1 dye (5 μM). In resting T cells, both TRPM8 agonist or antagonist decreases the membrane potential whereas in TCR‐treated cells the membrane potential increased in all condition (but in AMTB‐treated consition, the level increases significantly). Scale bar: 10 μm. Quantification of fluorescence intensities of ATP‐red (b, c) and JC1 (e, f) in resting and TCR‐treated cells. (*n* ≥ 100 cells in each condition were quantified, One‐way ANOVA, *p < 0.05, **p < 0.01, ***p < 0.001, ****p < 0.0001). # represents comparison with resting control cells (student's *t*‐test, ^#^ *p* < 0.05).

To investigate the function of TRPM8 in relation to ATP synthesis, ATP‐Red dye (which determines mitochondrial ATP level specifically) was used. In resting T cells, activation of TRPM8 by WS12 or Menthol did not alter the ATP levels. Conversely, the inhibition of TRPM8 by AMTB leads to higher ATP generation (Figure 7a,b). Similar to resting cells, TRPM8 activation in TCR‐treated T cells had minimal effect on ATP levels, although a slight increase was observed. However, TRPM8 inhibition led to a substantial elevation in ATP‐levels in TCR‐stimulated cells as well. Altogether, our data suggest that inhibition of TRPM8 results in an increment in ATP production, with TCR‐treated T cells exhibiting elevated ATP levels. This phenomenon may be associated with mitochondrial Ca^2+^ and cellular Ca^2+^‐load, as shown in AMTB‐treated cells and TCR‐treated cells, which have greater mitochondrial Ca^2+^ (Figure 7a,c).

### 3.6 TRPM8 Modulation Affects Mitochondrial Membrane Potential

As per the JC‐1 dye labelling, in resting conditions, WS12 or Menthol administration results in a reduction of mitochondrial membrane potential (ΔΨm) in T cells. AMTB‐mediated inhibition of TRPM8 exhibits a comparable effect. This suggests that in a controlled condition, the ΔΨm remains elevated, and any disruption of TRPM8 function (either by activation or by inhibition) adversely impacts the mechanisms necessary for sustaining elevated ΔΨm (Figure 7d,e). Basal ΔΨm is ~2.93‐fold higher in TCR‐treated T cells (as compared to the resting conditions). In TCR‐treated conditions, TRPM8 activation by WS12 or Menthol marginally lowers ΔΨm. Inhibition of TRPM8 by AMTB under TCR‐treated conditions results in the highest (~3.07‐fold) ΔΨm, suggesting that the endogenous activity of TRPM8 has a role in the maintenance of ΔΨm. Consequently, pharmacological inhibition of TRPM8 affects the T cells variably based on their immunological conditions (Figure 7d,f).

### 3.7 TRPM8 Modulation Affects Levels of Cardiolipin

**FIGURE 8:**
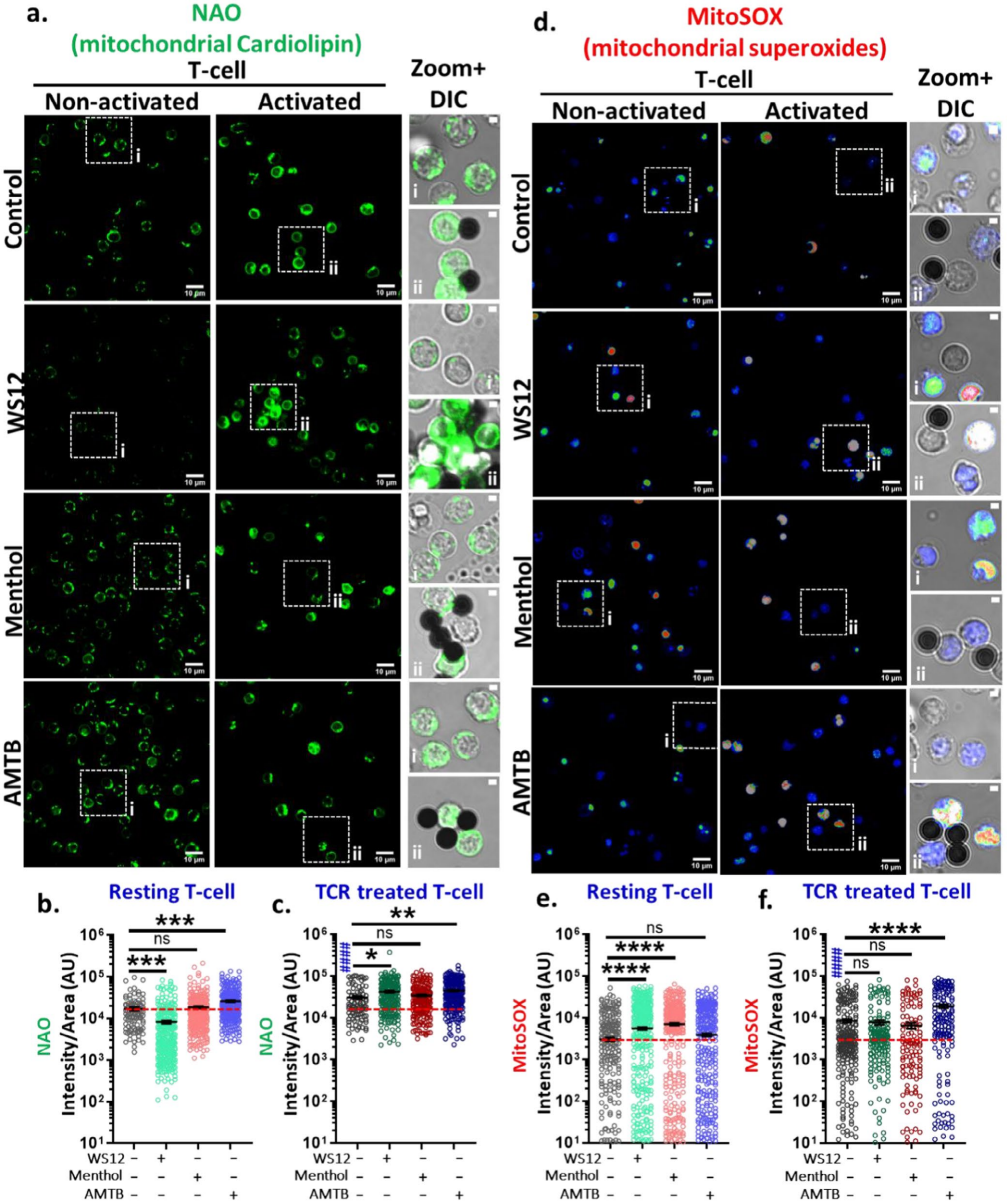
TRPM8 regulates mitochondrial cardiolipin and ROS levels. (a–c) Resting and TCR‐treated CD3^+^T cells were treated with TRPM8 agonist WS12 (5 μM), Menthol (50 μM) and TRPM8 antagonist AMTB (10 μM). The mitohcondrial cardiolipin was measured by using NAO (5 μM). Scale bar is 10 μm. (d–f) Both resting and TCR‐treated cells were treated with different TRPM8 modulators and subsequently stained with MitoSOX red (2.5 μM). In TCR‐treated cells, the ROS level elevated by the presence of AMTB. Scale bar is 10 μm. Quantification of fluoresence intensities of NAO (b, c) and MitoSOX (e, f) is represented. (*n* ≥ 100 cells in each condition, One‐way ANOVA, ns = *p* > 0.05 & non‐significant, *= *p* < 0.05, **p < 0.01, ***p < 0.001, ****p < 0.0001). # represents comparison with resting control cells (student's *t*‐test, ^####^ *p* < 0.0001).

Cardiolipin is a mitochondria‐specific phospholipid that plays a pivotal role in the regulation of mitochondrial structure and function in T cells [24]. This prompted us to evaluate the alteration in cardiolipin status in the T cells following the manipulation of TRPM8. NAO dye was employed to probe the levels of cardiolipin and its alterations (Figure 8a,c). NAO selectively binds to the mitochondrial phospholipids, functioning as a probe irrespective of membrane potential. It primarily detects the cardiolipins present in the mitochondria [25]. In resting T cells, activation of TRPM8 by WS12 reduces the cardiolipin levels (0.47‐fold). However, such reduction is not observed when TRPM8 is activated by Menthol (discussed later). Furthermore, AMTB, the TRPM8‐specific antagonist, elevates cardiolipin levels (~1.51‐fold) (Figure 8a,b). We also assessed the alterations in cardiolipin levels under TCR‐treated conditions. In TCR‐treated T cells, the basal level of cardiolipin is ~1.78‐fold higher than that of resting T cells. Moreover, in TCR‐treated conditions, further activation of TRPM8 by WS12 causes a further increment (1.34‐fold) in cardiolipin levels. However, in the TCR‐treated state, Menthol doesn't impact the cardiolipin levels. In contrast, under TCR‐treated conditions, inhibition of TRPM8 leads to additional elevation in cardiolipin levels. Altogether, the data suggest that TRPM8 inhibition results in increased cardiolipin levels. Also, activation of TRPM8 by WS12 affects cardiolipin levels variably in activated and resting T cells (Figure 8a,c).

### 3.8 Activation of TRPM8 Causes More Mitochondrial Superoxides in Resting T Cells

We investigated whether TRPM8 regulates mitochondrial superoxides (mtROS), which are crucial molecules that affect immunological response. To measure mtROS, we used MitoSOX dye [26] (Figure 8d,f). In resting condition, TRPM8 activation by either WS12 or Menthol leads to an elevation in mitochondrial ROS levels (~1.81 and ~2.28‐fold, respectively). Upon TRPM8 inhibition by AMTB, the mitochondrial ROS levels become comparable to resting conditions (Figure 8d,e). Under TCR‐treated conditions, the mitochondrial ROS level increases significantly (~2.79‐fold) compared to resting T cells. In TCR‐treated T cells, activation of TRPM8 does not augment mitochondrial ROS further. Inhibition of TRPM8 by AMTB results in an increase in mitochondrial ROS in activated T cells (~2.24‐fold) relative to resting T cells, suggesting that endogenous TRPM8 activity maintains low ROS levels in activated T cells (Figure 8d,f). Taken together, the endogenous activity of TRPM8 contributes to the mitochondrial ROS levels, especially in a context‐dependent manner.

### 3.9 TRPM8 Modulation Alters Mitochondrial Temperature

**FIGURE 9:**
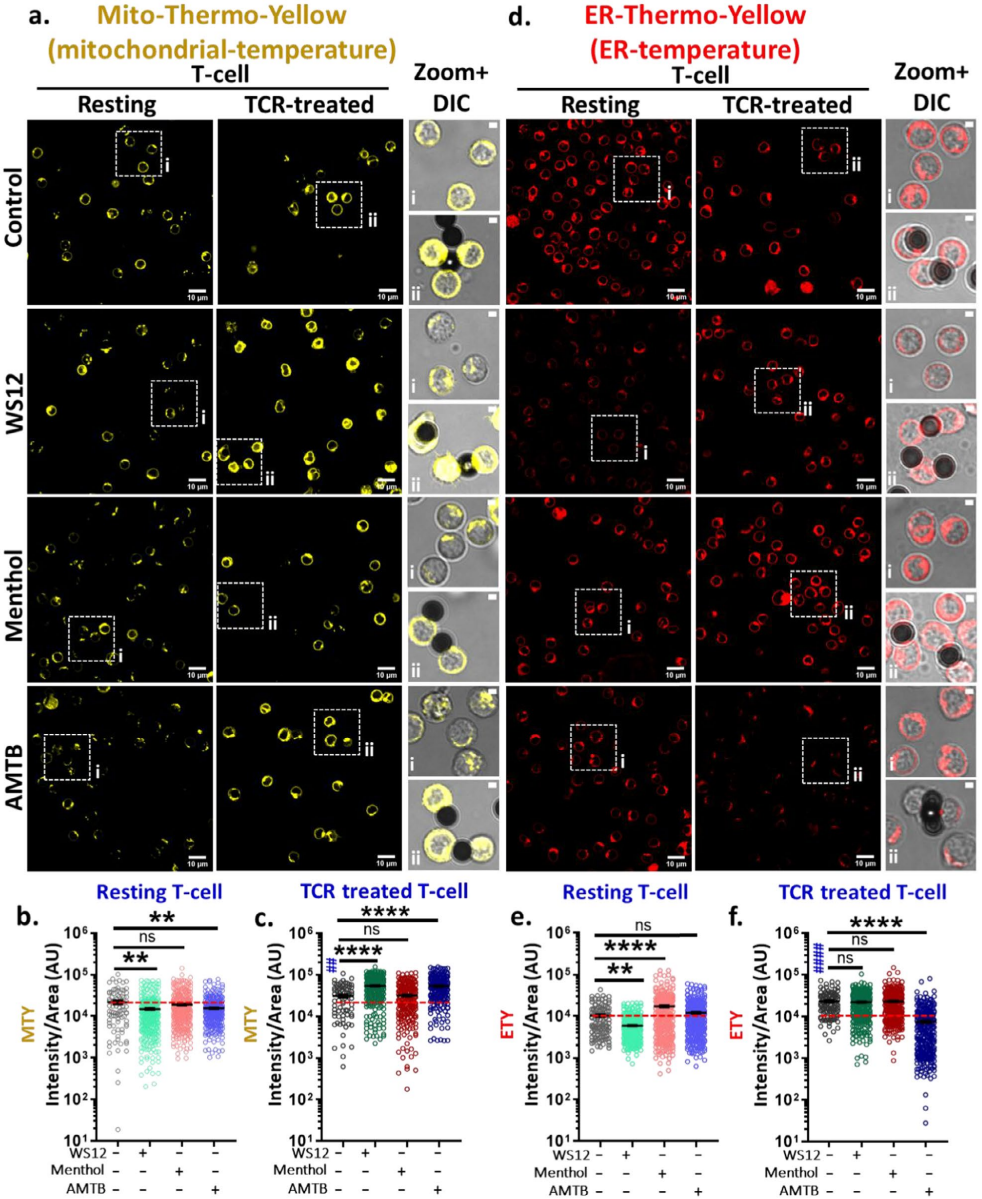
TRPM8 regulates mitochondrial and ER‐temperature. (a–c) Resting and TCR‐treated murine CD3^+^ T cells were treated with TRPM8 agonist WS12 (5 μM), Menthol (50 μM) and TRPM8 antagonist AMTB (10 μM). The mitochondrial temperature was measured by MTY (0.5 μM). In resting cells, the mitochondrial temperature increased by the addition of WS12 and AMTB (decrease in intensity). In TCR‐treated cells, the mitochondrial temperature decreased in WS12‐ and AMTB‐treatment (increase in intensity). Scale bar 10 μm. (d–f) Both resting and TCR‐treated cells were treated with different TRPM8 modulators and subsequently stained with ETY (0.5 μM). In resting T cells, the ER temperature was increased by WS12 whereas TRPM8 inhibition by AMTB does not change the ER temperature. In TCR‐treated cells, the ER‐temperature is increased (decrease in temperature) due to AMTB whereas the temperature remains unchanged in case of agonist treatments. Scale bar 10 μm. Fluorescence intensities of individual cells for MTY (b, c) and ETY (e, f) were represented in the graph (*n* ≥ 100 cells in each condition, One‐way ANOVA, **p < 0.01, ****p < 0.0001). # represents comparison with resting control cells (student's *t*‐test, ^##=^ *p* < 0.01, ^####^ *p* < 0.0001).

Mitochondrial temperature and also the change in temperature are important parameters that regulate mitochondrial metabolism, and thus the ATP production, which is a critical limiting factor for T cell activation. We used MTY dye to assess the TRPM8‐mediated alterations in mitochondrial temperature (Figure 9a–c). MTY is a molecular probe that inversely alters its fluorescence properties based on the mitochondrial temperature. In resting T cells, TRPM8 modulation leads to a decrease in MTY fluorescence intensity, indicating an increase in mitochondrial temperature. No change in mitochondrial temperature is observed by the addition of Menthol (Figure 9a,b). In TCR‐treated conditions, the mitochondrial temperature is reduced as indicated by increased fluorescence intensity of MTY as compared to resting T cells. However, in TCR‐treated conditions, activation by WS12 or inhibition by AMTB leads to an increase in MTY fluorescence (suggesting lowering of the mitochondrial temperature). In the TCR‐treated condition, Menthol has less impact (discussed later) (Figure 9a,c). Collectively, this suggests that TRPM8 affects the mitochondrial temperature, but the extent of such depends on the immunological state.

### 3.10 TRPM8 Activation Affects ER Temperature in Resting but Not in Activated T Cells

Mitochondria are physically and functionally connected with the ER, and such connectivity is especially required for the efficient exchange of different metabolites and Ca^+2^ [27]. We used ETY dye‐based fluorescence for evaluating the changes in the ER temperature by TRPM8 (Figure 9d–f). In resting T cells, WS12 reduces ETY fluorescence intensity, signifying an elevated ER temperature. Under the Menthol‐treated condition, the ETY fluorescence intensity increased (discussed later), while AMTB exhibited no effect (Figure 9d,e). In TCR‐treated T cells, the basal ETY intensity is elevated in comparison to resting T cells. These results demonstrate that the ER temperature is reduced in activated T cells relative to resting conditions. Under TCR‐treated conditions, further TRPM8 activation by either WS12 or Menthol doesn't alter ER temperature. In contrast, inhibition of TRPM8 by AMTB greatly elevates the ER temperature as the fluorescence intensity of ETY declines (Figure 9d,f). This suggests that spontaneous activity of TRPM8 maintains ER at low temperature.

### 3.11 TRPM8 Inhibition Increases ER‐Mitochondrial Contact Points

**FIGURE 10:**
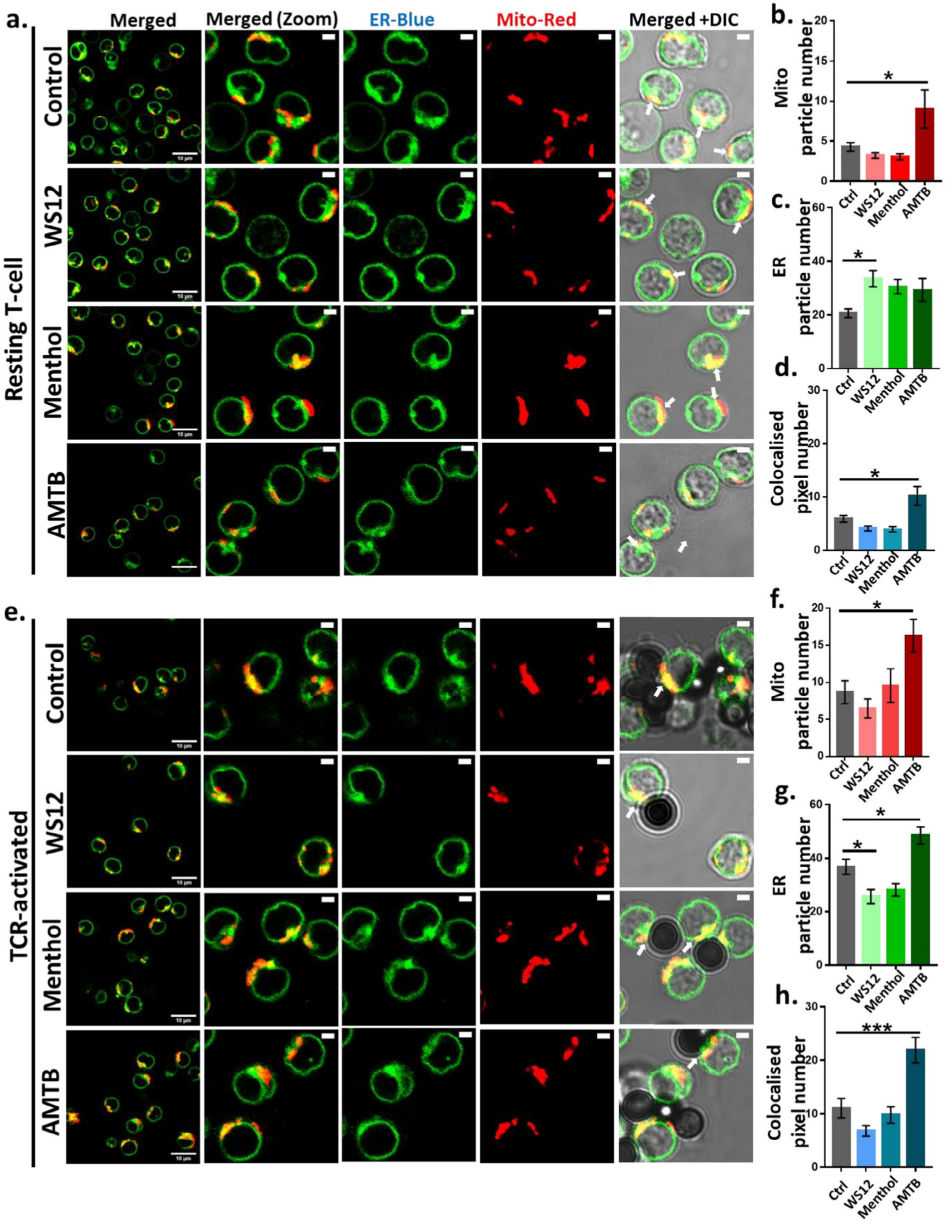
TRPM8 regulates ER‐mitochondrial contact sites. Resting (a–d) and TCR‐treated CD3^+^T cells (e–h) were treated with WS12 (5 μM), Menthol (50 μM), AMTB (10 μM), and was labelled by mitoCMXros (0.5 μM) and ER‐tracker blue (0.5 μM). The total mitochondrial, total ER and colocalized particles were quantified by ImageJ. (a–d) In resting T cell, the mitochondria particle increased in AMTB‐treated condition and the ER‐particle increased in WS12‐treated condition. The total colocalized particle number is increased at the AMTB‐treated cells. Scale bar 2 μm. (e–h) In TCR‐treated cells, both mitochondrial and ER‐particle numbers increased as compared to resting T cells. In this condition, the colocalized particle number increased significantly in the AMTB treated cells. Scale bar 2 μm. (*n* ≥ 30 cells in each condition were quantified). The quantified graphs are represented in the right side of the images. ER–Tracker blue is represened in pseudocolour green in the representative images. One‐way ANOVA, *p < 0.05, ***p < 0.001. # represents comparison with resting control cells (student's *t*‐test, ^##^ *p* < 0.01, ^####=^ *p* < 0.0001).

ER‐mito contact points are essential for effective Ca^2+^‐buffering and thus for maintaining Ca^2+^‐homeostasis [27]. ER‐mitochondrial contact sites are crucial for the exchange of different ions and metabolites. The flow rate of metabolites from ER and mitochondria is contingent upon the existing relative thermal gradient between these two compartments, as well as the extent of ER‐mitochondrial contact areas [28, 29]. We investigated whether TRPM8 modulation affects the ER‐mitochondrial contacts. For that purpose, we performed particle‐based analysis and quantified total mitochondria, the total ER and total colocalized particles (Figure 10a–h). Under resting conditions, the administration of WS12 or Menthol does not alter the number of mitochondria particles but increases the ER particles. Under resting conditions, the inhibition of TRPM8 by AMTB increases (~2.1‐fold) the number of mitochondria significantly as compared to untreated cells. This correlates well with the increased ER‐mitochondrial colocalization following TRPM8 inhibition (~1.72‐fold) (Figure 10a–d). A comparable trend is noted in the TCR‐treated condition, where the activation of TRPM8 doesn't elicit any significant changes. However, in the TCR‐treated condition, TRPM8 inhibition results in the highest number of mitochondrial particles, which is even higher than resting T cells treated with AMTB. This also accords well with the fact that TRPM8 inhibition causes ~2‐fold more ER‐mitochondrial contact points. Under TCR‐treated conditions, the application of WS12 or Menthol leads to a reduction in ER particle numbers, whereas AMTB elevates the number of ER particles (~1.32‐fold). We noted that the mitochondrial particles are higher in TCR‐treated conditions than in resting T cells, and this is irrespective of TRPM8 modulation. The total ER particles are higher in the TCR‐treated condition compared to the resting T cell (Figure 10e–h).

## 4 Discussion

Previously, we reported that the surface expression of TRPM8 in T cells is elevated upon activation by Con A [12, 30]. During the T cell activation process, the majority of mitochondria translocate to the immediate vicinity of the IS [2]. Translocation of mitochondria to the IS not only regulates high Ca^2+^‐level at the IS, but also supplies higher ATP to mediate the T cell activation process and cytokine production [5]. So far, the physical localization of a few TRP ion channels in mitochondria has been reported. We have recently demonstrated the presence of TRPV4, a thermosensitive ion channel in mitochondria, where it modulates mitochondrial functions and temperature [14, 31, 32]. We have also identified that TRPV4 localises to the mitochondria of T cells and regulates mitochondrial physiology in both resting and in TCR‐treated T cells [14]. Likewise, a truncated 4TM‐TRPM8 localises at the MAM (Mitochondria Associated Membrane) where it regulates Ca^2+^‐status in both mitochondria and in ER [13]. In this study also, we noted the presence of TRPM8 in the mitochondrial enriched regions in naïve CD3+ T cells. Such localization may help regulate mitochondrial Ca^2+^ and other functionalities, which in turn helps in regulating naïve CD3 T cell functions and proliferations in the presence/absence of antigens. Mitochondrial Ca^2+^‐uptake is essential for cellular Ca^2+^‐signalling, and is dependent on cytosolic and store‐operated Ca^2+^‐sources (primarily from the ER). This study demonstrates that in T cells, TRPM8 regulates Ca^2+^‐influx in both cytosol as well as in mitochondria. Activation of TRPM8 elevates both cytosolic and mitochondrial Ca^2+^‐levels and regulates the close proximity between mitochondria and ER, which facilitates Ca^2+^‐import during the process of TCR‐mediated T cell activation. TRPM8 might act as one of the Ca^2+^‐ and Zn^2+^‐transporters inside the T cells, which can help the T cells proliferate and differentiate [33].

We evaluated that the Δ‐change in mitochondrial Ca^2+^, which is enhanced in TCR‐treated T cells upon TRPM8 agonist treatment. Data suggest that TRPM8‐mediated Ca^2+^‐influx is from both the extracellular and intracellular Ca^2+^‐sources. This is justified by the increase in the cellular Ca^2+^‐levels upon TRPM8 activation even under EGTA and BAPTA‐AM‐treated conditions. We explored further the impact of ER (as a Ca^2+^‐store) on mitochondrial Ca^2+^‐levels. We analysed the ΔCa^2+^‐values in Thapsigargin (a SERCA inhibitor)‐treated cells. We observed an increase in mitochondrial Ca^2+^‐levels with prolonged exposure to Thapsigargin followed by specific activation of TRPM8 by WS12. This observation suggests that WS12 stimulates TRPM8 present at the MAM region and elevates mitochondrial Ca^2+^. For effective Ca^2+^‐mobilisation into the mitochondria, MAM plays a crucial role, and our data indicate the changes in the extent of ER‐mitochondrial contact sites during TCR activation. We demonstrate that during the TCR‐activation, mitochondria make close contact with the ER, and these contact points are highly regulated by TRPM8 modulation. Notably, the number of ER‐mitochondrial contact points is highly dependent on mitochondrial number instead of ER number. This data accords well with our previous reports where we have shown that the mitochondrial Ca^2+^‐load is one of the determining factors of the establishment of [14, 31].

T cell proliferation is a highly energy‐dependent process [5]. In general, mitochondria act as “the source,” and the ER, as well as other sub‐cellular organelles, act as the “sink” for ATP. Our data also suggest an increase in mitochondrial ATP levels in TCR‐activated cells. Our data indicate that decreased mitochondrial Ca^2+^‐level correlates well with the reduced mitochondrial ATP levels, which seems to affect T cell activation. This accords well with the fact that mitochondrial ATP is greatly affected by the level of mitochondrial Ca^2+^‐load [34]. Pharmacological alteration of TRPM8 has no effect on the change in mitochondrial ATP levels during T cell activation. TRPM8 seems to provide an additive benefit for T cells to enhance mitochondrial respiration, followed by ATP generation.

Activation of TCR by CD3/CD28 co‐stimulation induces mitochondrial hyperpolarization by elevating mitochondrial membrane potential. The alteration in membrane potential is predominantly affected by cytosolic and mitochondrial Ca^2+^‐level [34]. Our findings align with the observation that TCR‐activation enhances mitochondrial membrane potential. We observed that in TCR‐treated cells, the mitochondria have higher Ca^2+^‐levels. There are reports that support relationships between mitochondrial Ca^2+^ and mitochondrial membrane potential [35].

Apart from mitochondrial metabolism, mitochondrial membrane lipid composition and integrity are required for effective T cell activation. Recently we have reported that TRPM8 inhibition reduces the size but increases the number of lipid droplets in mature adipocytes [36]. We also identified that TRPM8 residues are co‐evolved with cholesterol interaction at the lipid water interface of the membrane and are also involved in “heating‐up” as well as “cooling‐down” of the subcellular organelles [37]. Cardiolipin is a suitable phospholipid that is exclusively synthesised and localized at the inner mitochondrial membrane (it accounts for 15%–20% of total mitochondrial phospholipids) [24]. Cardiolipin is also important for T cell activation, as T cells deficient in cardiolipin respond poorly to T cell activation [24]. Here we report that overall mitochondrial cardiolipin levels are elevated in the TCR‐treated condition, and this reach a higher level in the WS12‐treated condition. This finding accords well with the fact that WS12 promotes T cell activation [12].

Mitochondrial ROS are produced by complex I, II and III of the electron transport chain [38]. During T cell activation, the mtROS level rises, mostly mediated by Ca^2+^‐influx [26]. We observed that TRPM8 activation elevates mitochondrial ROS during TCR activation. Consequently, we propose that TRPM8 regulates the T cell activation process by maintaining mitochondrial oxidative metabolism.

Increased core body temperature is associated with the activation of the innate immune system. Febrile‐ range temperatures selectively induce mitochondrial dysfunction and apoptosis in certain T cell subsets, particularly T helper 1 (Th1) cells. When mitochondria are exposed to fever temperatures (39°C), impairs the electron transport chain complex I, triggers mitochondrial DNA damage, and increases apoptosis in Th1 cells. As a result these temperature shifts have profound effects on immune response during infection, autoimmune diseases [39]. Cancer cells often upregulate mitochondrial uncoupling proteins, increasing mitochondrial temperature and metabolic rate [40]. In patients with mitochondrial diseases the brain show altered ability to regulate temperature and the brain is hypothermic due to dysfunction in oxidative phosphorylation [41]. Minor fluctuations in body temperature render thermosensitive TRP channels as viable candidates pertinent to immune regulation. In the cellular level, mitochondria are maintained at ~10°C hotter than the cytoplasm [42]. We identified that activation of TRPM8 by WS12 elevates the mitochondrial and ER temperature in resting T cells, whereas in TCR‐treated cells, it decreases the mitochondrial and ER temperature. It can be postulated that the thermoregulation at the sub‐cellular organelle level in T cells is a context‐dependent mechanism, which is also associated with the effective synthesis and trafficking of specific membrane proteins, signalling molecules, Ca^2+^, ATP, etc., required for T cell activation. As per our recent report modulation of TRPM8 alters transferrin‐mediated iron uptake into the mitochondria of the microglia [43]. These findings reveal that under specific conditions, TRPM8 modulation might lead to differential temperature regulation between ER and mitochondria, a mechanism that may be crucial for the regulation of the “thermal gradient” within the cell, and relevant for the diffusion of metabolites. Corroborating this finding, we recently described that menthol reduces the mitochondrial temperature in different cell types and in primary mesenchymal stem cells [16]. The results described in this work also accord well with our recent findings demonstrating that TRPM8 alters the temperature of sub‐cellular organelles in a context‐dependent manner [37]. However, the mechanistic involvement of TRPM8 in the regulation of subcellular organelle temperature needs further and detailed research.

We observed that in numerous instances, WS12 and menthol exhibit very differently. The disparity between these two agonists might originate from their chemical properties and/or global action. The dose needed of menthol (50 μM) to activate TRPM8 is significantly higher than WS12 (5 μM). Although menthol is widely used as a TRPM8 agonist, it is a pleiotropic compound that also modulates multiple other ion channels and receptors and can alter membrane biophysical properties. Therefore, menthol‐induced effects cannot be attributed exclusively to TRPM8 and may involve TRPM8‐independent mechanisms depending on cell context and channel localization [44, 45]. Although TRPM8 represents a cold‐activated ion channel and the ligands utilised in this study are exogenous, it is important to note that some endogenous ligands (such as 3‐Iodothyronamine) can interact with TRPM8 [46]. TRPM8 distribution, especially the surface localization, is also subject to regulation by membrane cholesterol and has an evolutionary history in its sequence [47].

**FIGURE 11:**
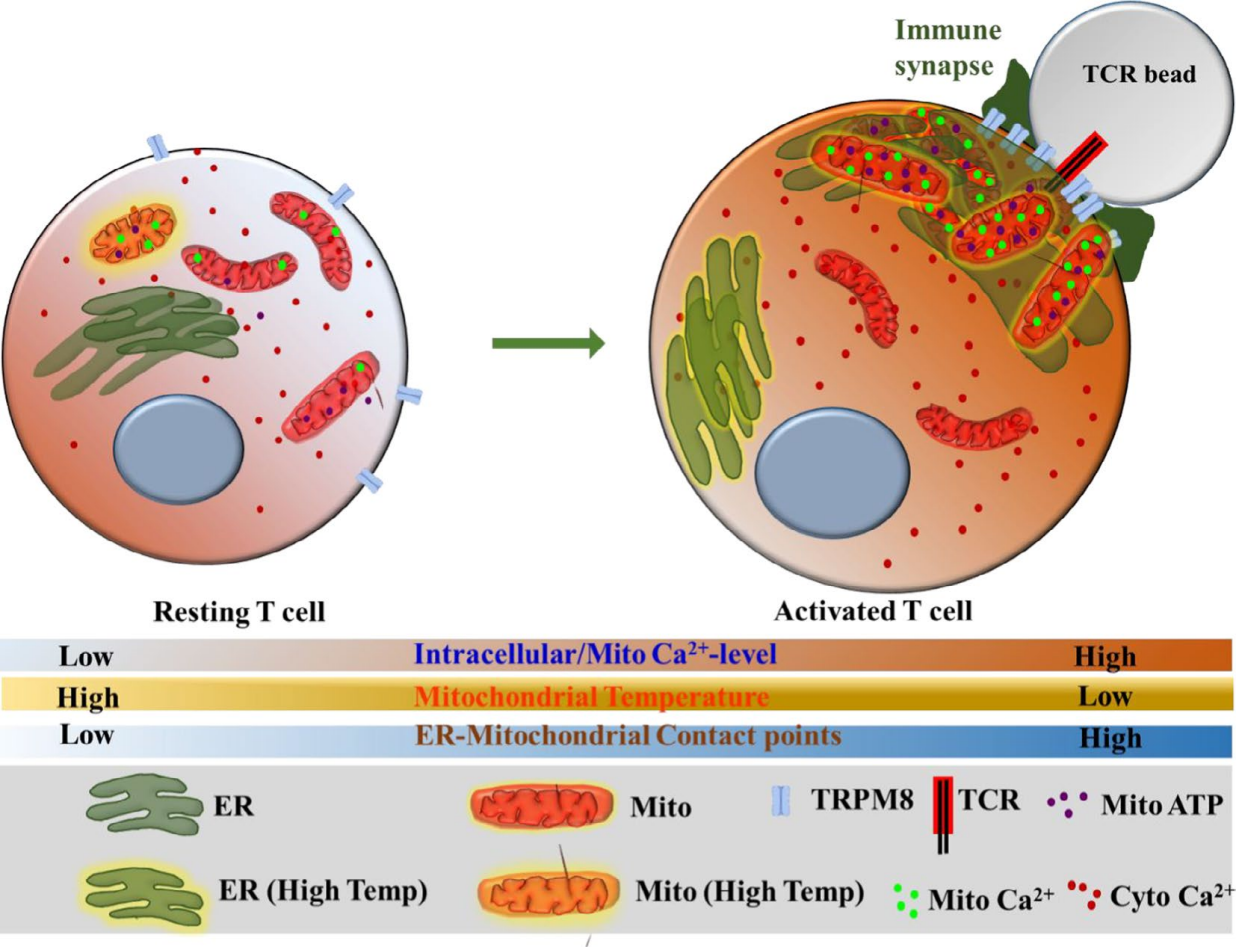
Proposed model illustrating the involvement of TRPM8 in mitochondrial function and metabolism in T cells. Functional TRPM8 channels seem to be localised in the mitochondria. TCR activation causes mitochondrial translocation to the IS. By activating the channel by synthetic and natural agonists, the mitochondrial Ca^2+^‐load increases, which in turn channels the Ca^2+^ into the cytosol for effective TCR‐mediated T cell activation and effector response. In the IS, the mitochondria increase ATP and ROS levels which play a prime role in T cell activation. TRPM8 modulation also regulates mitochondrial and ER temperature. In TCR‐treated cells, the mitochondrial number increases, which drives the mitochondria to make close contact with the ER, mediating an increase in ER‐mitochondrial contact points.

Our data suggests that both TRPV4 and TRPM8 contribute to multiple cellular functions. Notably, both channels regulate mitochondrial Ca^2+^‐load and also regulate mitochondrial/cytosolic Ca^2+^ ratio, the parameters which are relevant for TCR activation. This suggests that the mitochondrial Ca^2+^ is of utmost importance in T cell activation, rather than cytosolic Ca^2+^ only, and this phenomenon could be universal. Collectively, our findings indicate that indeed TRPM8 is localised at the T cell immunological synapse and regulates mitochondrial and ER physiology differentially in resting and TCR‐activated conditions (Figure 11).

Our findings underscore a novel paradigm in which TRPM8 fine‐tunes T cell immune responses by integrating bioenergetics and metabolic reprogramming mediated by mitochondria and thermal homeostasis at the subcellular level. Considering the centrality of T cell activation in autoimmunity, inflammation and cancer, targeting mitochondrial TRPM8 presents a promising therapeutic avenue for immunomodulation. Our study therefore expands the scope of TRPM8 beyond classical sensory roles and establishes it as a potential molecular target for translational strategies aimed at regulating T cell‐mediated immunity.

Our study provides functional evidence for TRPM8‐mediated regulation of mitochondrial Ca^2+^ dynamics, temperature and ER‐mitochondria contacts; however, direct biochemical evidence of TRPM8 localization within T cell mitochondria remains to be established. Further detailed investigation is required to conclusively establish TRPM8 localization in the mitochondrial membrane and the mechanistic basis of TRPM8‐mediated mitochondrial thermoregulation.

## Author Contributions

C.G. conceptualised the project, provided reagents, facilities for the experiments and provided guidance. T.K.A., S.K., T.P.R., P.M. performed all the experiments and analysed the data. P.M. performed the super‐resolution imaging. Y.T.C. provided the ETY, MTY and ATP‐Red dyes. C.G. critically analysed the data. T.K.A, S.K., T.P.R., P.M. and C.G. compiled the figures for the manuscript. All authors contributed to the manuscript preparation. C.G. edited the final manuscript and finalised the author list. All authors have gone through the final manuscript and endorse the manuscript submission.

## Conflicts of Interest

The authors declare no conflicts of interest.

## Data Availability

The data that support the findings of this study are available from the corresponding author upon reasonable request.
